# N-Heterocyclic Carbene Modified Palladium Catalysts for the Direct Synthesis of Hydrogen Peroxide

**DOI:** 10.1021/jacs.2c04828

**Published:** 2022-08-17

**Authors:** Richard J. Lewis, Maximilian Koy, Margherita Macino, Mowpriya Das, James H. Carter, David J. Morgan, Thomas E. Davies, Johannes B. Ernst, Simon J. Freakley, Frank Glorius, Graham J. Hutchings

**Affiliations:** † Max Planck Cardiff Centre on the Fundamentals of Heterogeneous Catalysis, FUNCAT, Cardiff Catalysis Institute, School of Chemistry, Cardiff University, Main Building, Park Place, Cardiff CF103AT, United Kingdom; ‡ Westfälische Wilhelms-Universität Münster, Organisch-Chemisches Institut, Corrensstraße 36, 48149 Münster, Germany; § Harwell XPS, Research Complex at Harwell (RCaH), Didcot OX110FA, United Kingdom; ∥ Department of Chemistry, University of Bath, Claverton Down, Bath BA27AY, United Kingdom

## Abstract

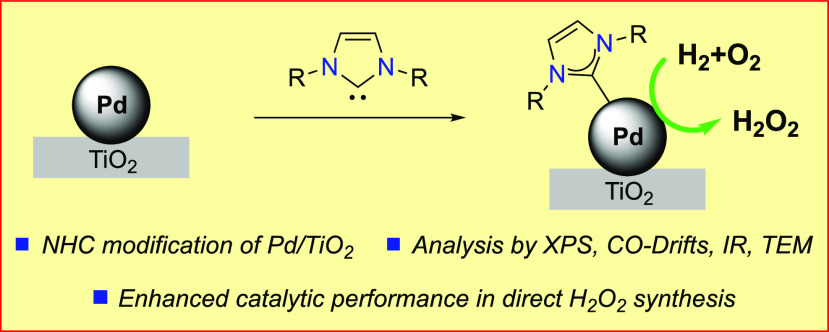

Heterogeneous palladium catalysts modified by N-heterocyclic carbenes (NHCs) are shown to be highly effective toward the direct synthesis of hydrogen peroxide (H_2_O_2_), in the absence of the promoters which are typically required to enhance both activity and selectivity. Catalytic evaluation in a batch regime demonstrated that through careful selection of the N-substituent of the NHC it is possible to greatly enhance catalytic performance when compared to the unmodified analogue and reach concentrations of H_2_O_2_ rivaling that obtained by state-of-the-art catalysts. The enhanced performance of the modified catalyst, which is retained upon reuse, is attributed to the ability of the NHC to electronically modify Pd speciation.

N-Heterocyclic carbenes (NHCs) are well-established compounds in various fields of chemistry and find application as ligands for numerous processes in the field of homogeneous catalysis.^1−3^ This is due to their effective and controllable donor capability and highly modular structure. As a result, parameters such as stability, reactivity, and selectivity can be effectively tuned, allowing ligands to be tailored for a wide variety of applications. Well-known precatalysts containing NHCs as ligands include PEPPSI (pyridine-enhanced precatalyst preparation stabilization and initiation) and the second-generation Grubbs(−Hoveyda) catalysts.^4,5^ In comparison, the systematic application of NHCs as ligands in heterogeneous catalysis is still in its infancy.^6,7^ There is a growing number of reports using NHCs for surface modification,^8−10^ with many studies demonstrating their ability to control important catalytic properties (Figure 1A presents an example of both an NHC-based homogeneous catalyst and an NHC-promoted heterogeneous catalyst).^11−46^ Building on these fundamental discoveries, the goal of this work is to use NHC-modified heterogeneous catalysts for the production of the commodity chemical hydrogen peroxide (H_2_O_2_) from the elements (Figure 1B).

**Figure 1:**
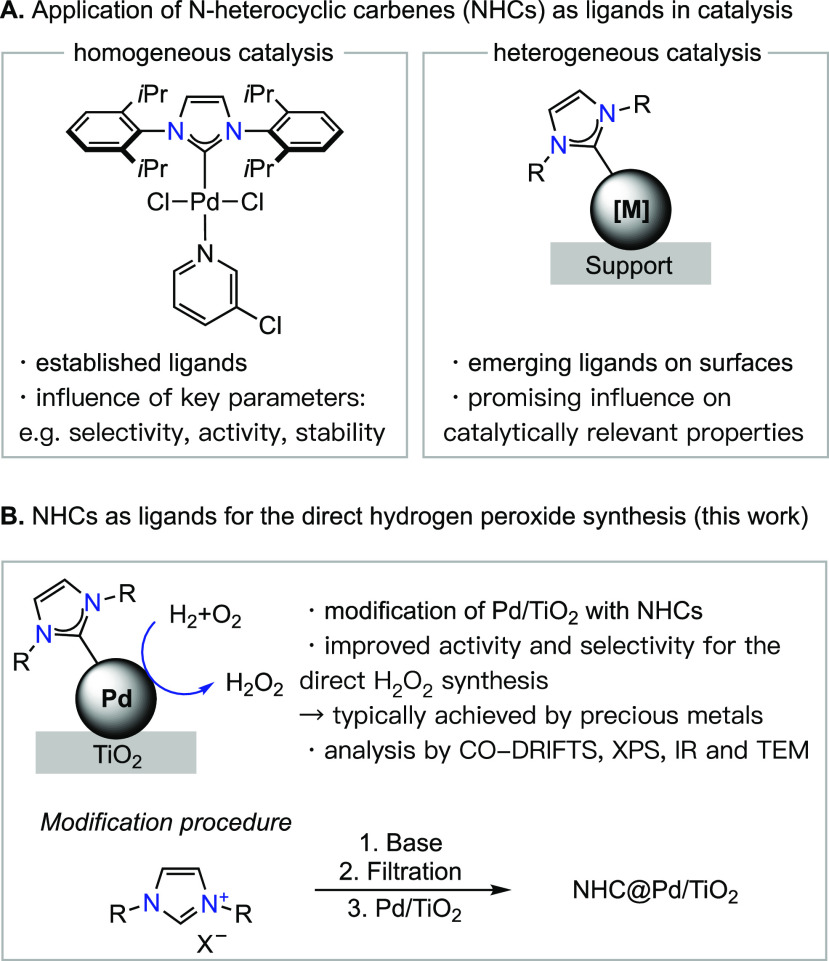
N-Heterocyclic carbenes as ligands in catalysis and their application for the direct synthesis of hydrogen peroxide.

Global demand for H_2_O_2_ has risen significantly in recent years, driven largely by its use as an oxidant for a range of chemical transformations, as well as its utilization as a bleaching agent. Currently, industrial production of H_2_O_2_ is met almost entirely via the highly efficient anthraquinone oxidation process.^19^ However, numerous routes to small-scale H_2_O_2_ production have been investigated, including electrochemical,^20^ photocatalytic,^21^ and thermal catalytic approaches.^22^ The thermal catalytic direct synthesis of H_2_O_2_ from the elements is considered particularly attractive for on-site production, at desirable concentrations of this powerful oxidant, and theoretically allows for total atom efficiency. The direct route is of particular interest for chemical processes where the generated H_2_O_2_ is utilized *in situ* for chemical valorization^23^ or pollutant degradation.^24^ Pd-based catalysts have been widely studied for the direct synthesis reaction;^25^ however, they typically suffer from poor selectivity and require the use of halide^26−28^ or acid promoters.^29^ While the use of such agents can significantly enhance catalytic performance, their application can have deleterious effects on catalyst and reactor lifetimes and lead to the formation of complex product streams. Indeed, in the case of some catalyst formulations the use of halide additives can lead to a near-total inhibition of catalytic performance;^30^ as such, there is a need for alternative approaches to improve catalytic activity and selectivity.

The introduction of secondary metals^31−34^ has also been demonstrated to inhibit competitive reaction pathways while avoiding the need for the stabilizing agents typically utilized for Pd-only analogues. Notably, Fischer et al. have reported that combining the stabilizing agents typically utilized for Pd-only catalysts (HBr and H_3_PO_4_) with bimetallic Pd-based formulations exceptionally high concentrations of H_2_O_2_ can be obtained. It should be emphasized that such concentrations are far greater than those often reported in the literature and are even more remarkable, given the use of a water-only reaction medium, which avoids the additional process costs associated with the alcohol cosolvents commonly used to promote H_2_ solubility.^35^ However, the additional costs and often complex synthesis procedures associated with the use of bimetallic catalysts have prompted a focus on alternative means to improve the performance of Pd-only formulations. The encapsulation of supported Pd nanoparticles in organic moieties such as poly(vinyl alcohol) or poly(vinylpyrrolidone) has been shown to enhance performance by selectively tuning the three-dimensional environment of the metal nanoparticle.^36−38^ Recently, the groups of Pérez-Ramírez and Nikolla have expanded on these studies, with the latter establishing the efficacy of a series of surface-bound ligand modifiers to promote the selectivity of Pd nanoparticles toward H_2_O_2_.^39,40^

Herein, the effect of a range of NHCs (ICy, IMes, IPr, *p*Ph-IPr, and IPr*) on the catalytic performance of supported Pd catalysts^41^ toward the direct synthesis and subsequent degradation of H_2_O_2_ was investigated. For this purpose, the free NHCs were prepared via deprotonation of the corresponding imidazolium salts and subsequently immobilized onto a 1%Pd/TiO_2_ catalyst. Successful NHC deposition onto the catalyst surface was confirmed using attenuated total reflectance infrared spectroscopy (ATR-IR) (Figure S.1) and corroborated by XPS (Figure S.2; corresponding spectra of the imidazolium salts are reported in Figure S.3). Initial catalytic testing established the activity of the NHC-modified 1%Pd/TiO_2_ catalysts (Table 1). The unmodified 1%Pd/TiO_2_ catalyst (entry 1) was found to be highly active toward H_2_O_2_ synthesis (80 mol_H2O2_ kg_cat_^–1^ h^–1^) but also displayed considerable activity toward its subsequent degradation (221 mol_H2O2_ kg_cat_^–1^ h^–1^). The introduction of the various NHCs (so that the Pd:NHC molar ratio was equal to 1:1) was found to greatly modify catalytic activity toward both the direct synthesis and subsequent degradation of H_2_O_2_. In particular, the optimal 1%Pd-IPr(1:1)/TiO_2_ catalyst (entry 4) offered H_2_O_2_ synthesis rates (160 mol_H2O2_ kg_cat_^–1^ h^–1^) double that of the unmodified analogue, while degradation rates were reduced (184 mol_H2O2_ kg_cat_^–1^ h^–1^). Indeed, the catalytic activity of the 1%Pd-IPr(1:1)/TiO_2_ catalyst can be considered to rival that achieved by state-of-the-art materials,^31,32^ under identical reaction conditions, although it should be noted that the NHC-modified material is unable to attain the high selectivities toward H_2_O_2_ such as those reported in earlier works (Table S.1). The improved activity of the 1%Pd-IPr(1:1)/TiO_2_ catalyst was also observed under conditions considered less conducive to H_2_O_2_ stability (Table S.2). Further studies, comparing the activity of the optimal 1%Pd-IPr(1:1)/TiO_2_ catalyst to an equimolar physical mixture of 1%Pd/TiO_2_ and imidazolium salt (IPr-HBF_4_) (entry 7), indicated that the NHC must be present on the catalytic surface in order to achieve enhanced activity toward H_2_O_2_. It should be noted that neither the IPr-HBF_4_ salt alone nor the titania support exhibited any activity toward H_2_O_2_ synthesis or its subsequent degradation (entries 8 and 9, respectively).

**Table 1:**
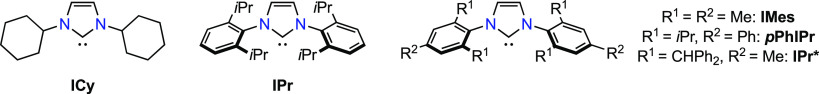
Influence of Structurally Diverse NHCs on the Productivity and Selectivity of 1%Pd/TiO_2_ toward the Direct Synthesis of H_2_O_2_

entry	catalyst	productivity/mol_H2O2_ kg_cat_^–1^ h^–1^	H_2_O_2_ concn/wt %	apparent rate of reaction at 30 min/mmol_H2O2_ mmol_Pd_^–1^ min^–1^	degradation/mol_H2O2_ kg_cat_^–1^ h^–1^
1	1%Pd/TiO_2_ (unmodified)	80	0.16	8.73 × 10^2^	231
2	1%Pd-ICy(1:1)/TiO_2_	110	0.21	1.19 × 10^3^	208
3	1%Pd-IMes(1:1)/TiO_2_	133	0.27	1.40 × 10^3^	202
4	1%Pd-IPr(1:1)/TiO_2_	160	0.32	1.70 × 10^3^	184
5	1%Pd-*p*Ph-IPr(1:1)/TiO_2_	118	0.23	1.26 × 10^3^	169
6	1%Pd-IPr*(1:1)/TiO_2_	110	0.21	1.15 × 10^3^	137
7	1%Pd/TiO_2_ + IPr-HBF_4_	79	0.16	8.41 × 10^2^	237
8	IPr-HBF_4_	0	0	0	0
9	TiO_2_ (P25)	0	0	0	0

The stark improvement in catalytic performance observed over the 1%Pd-IPr(1:1)/TiO_2_ catalyst, in comparison to the unmodified analogue, motivated us to further investigate the effect of varying the Pd:IPr molar ratio on catalytic performance (Figure 2A). These studies indicated an optimal catalyst composition of 1%Pd-IPr(1:1)/TiO_2_. Increasing IPr content further was found to result in a decrease in H_2_O_2_ production, with a corresponding increase in H_2_O_2_ degradation, although it is noteworthy that, despite this loss in catalytic selectivity at higher IPr loadings, all IPr-containing catalysts still outperformed the 1%Pd/TiO_2_ analogue.

**Figure 2:**
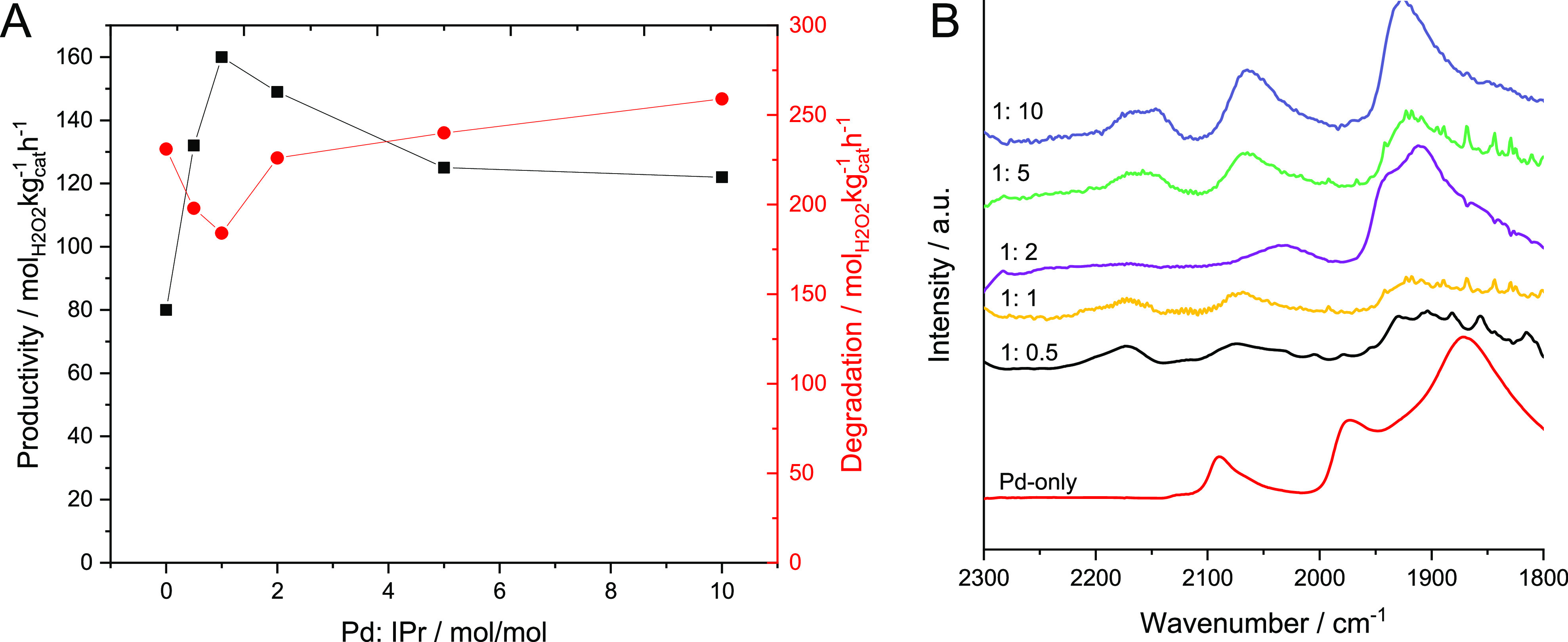
Effect of Pd:IPr ratio on catalytic performance toward the direct synthesis of H_2_O_2_. (A) Catalytic activity of NHC-modified 1%Pd/TiO_2_ catalysts toward the direct synthesis of H_2_O_2_ and its subsequent degradation as a function of Pd:IPr molar ratio. (B) CO-DRIFTS spectra of the 1%Pd-IPr/TiO_2_ catalysts, as a function of Pd:IPr molar ratio. H_2_O_2_ direct synthesis reaction conditions: catalyst (0.01 g), H_2_O (2.9 g), MeOH (5.6 g), 5% H_2_/CO_2_ (420 psi), 25% O_2_/CO_2_ (160 psi), 0.5 h, 2 °C, 1200 rpm. H_2_O_2_ degradation reaction conditions: catalyst (0.01 g), H_2_O_2_ (50 wt %, 0.68 g) H_2_O (2.22 g), MeOH (5.6 g), 5% H_2_/CO_2_ (420 psi), 0.5 h, 2 °C, 1200 rpm.

With NHCs well-known to act as modifiers of metal species, we next set out to determine the means by which the catalytic performance was enhanced through NHC incorporation. The evaluation of the 1%Pd-IPr/TiO_2_ catalysts with varying Pd:IPr molar ratio by CO-DRIFTS is shown in Figure 2B (an analysis of the IPr/TiO_2_ material (i.e., without Pd present) is reported in Figure S.4). Typically, the CO-DRIFTS spectra of supported Pd catalysts include CO adsorbed in a linear and nonlinear mode at approximately 2050–2100 and 1800–2000 cm^–1^, respectively.^42^ The 1%Pd/TiO_2_ catalyst was found to exhibit the expected absorption bands, specifically at 2090, 1980, and 1870 cm^–1^. The addition of the IPr moiety results in two major changes to the CO-DRIFTS spectra. This includes a new absorption band, which appears at 2170 cm^–1^, suggesting a new adsorption site associated with the IPr-containing catalysts.

A systematic shift in the wavenumber of the linear CO–Pd band was observed as the IPr:Pd molar ratio increased, from 2090 cm^–1^ in the 1%Pd/TiO_2_ catalyst to 2060 cm^–1^ in the 1%Pd-IPr(1:10)/TiO_2_ formulation. Such a shift indicates that the adsorption of CO onto the Pd surface increases in strength, which can be explained by the transfer of charge from the NHC to the Pd surface and the resulting enhanced back-donation to CO. Similar observations have been made by Ouyang et al., who reported a comparable red shift upon the alloying of Au with Pd and an associated enhancement in catalytic selectivity.^43^ It is therefore possible to conclude the enhanced activity that results from the introduction of the NHCs onto the catalyst surface can be attributed to the ability of the carbene moiety to electronically modify Pd species.

Finally, with the evident improved efficacy of the 1%Pd-IPr(1:1)/TiO_2_ catalyst in comparison to the unmodified 1%Pd/TiO_2_ analogue established, we were motivated to investigate this subset of materials to gain further insight into the underlying cause for the observed differences in performance. An assessment of selectivity toward H_2_O_2_ (Table S.3) further demonstrates the improvement that results from the introduction of the carbene onto the catalytic surface, with the 1%Pd-IPr(1:1)/TiO_2_ catalyst displaying far greater selectivity toward H_2_O_2_ (64%) than the 1%Pd/TiO_2_ analogue (22%). In keeping with earlier studies (Table 1, entry 7) an evaluation of fresh and used materials by XPS indicates that the observed promotive effect that results from NHC incorporation cannot be attributed to the presence of residual halide (Table S.4). The enhanced activity of the 1%Pd-IPr(1:1)/TiO_2_ catalyst was further highlighted through a comparison of initial reaction rates (Table S.5), where there are assumed to be no limitations associated with reactant availability or contribution from H_2_O_2_ degradation pathways.

A comparison of catalytic activity as a function of reaction time can be seen in Figure 3A, with the greater activity of the 1%Pd-IPr(1:1)/TiO_2_ catalyst again clear, achieving concentrations of H_2_O_2_ (0.32 wt %) double that of the unmodified analogue (0.16 wt %), over a standard 0.5 h reaction. Indeed, the 1%Pd-IPr(1:1)/TiO_2_ catalyst displayed rates of H_2_O_2_ synthesis comparable to those reported for the current state-of-the-art materials.^31,32^ A further evaluation of catalytic performance over sequential H_2_O_2_ synthesis reactions (Figure 3B) again demonstrates the enhanced activity of the 1%Pd-IPr(1:1)/TiO_2_ catalyst, which was able to achieve H_2_O_2_ concentrations (0.98 wt %) comparable to that achieved by state-of-the-art materials.^32^

**Figure 3:**
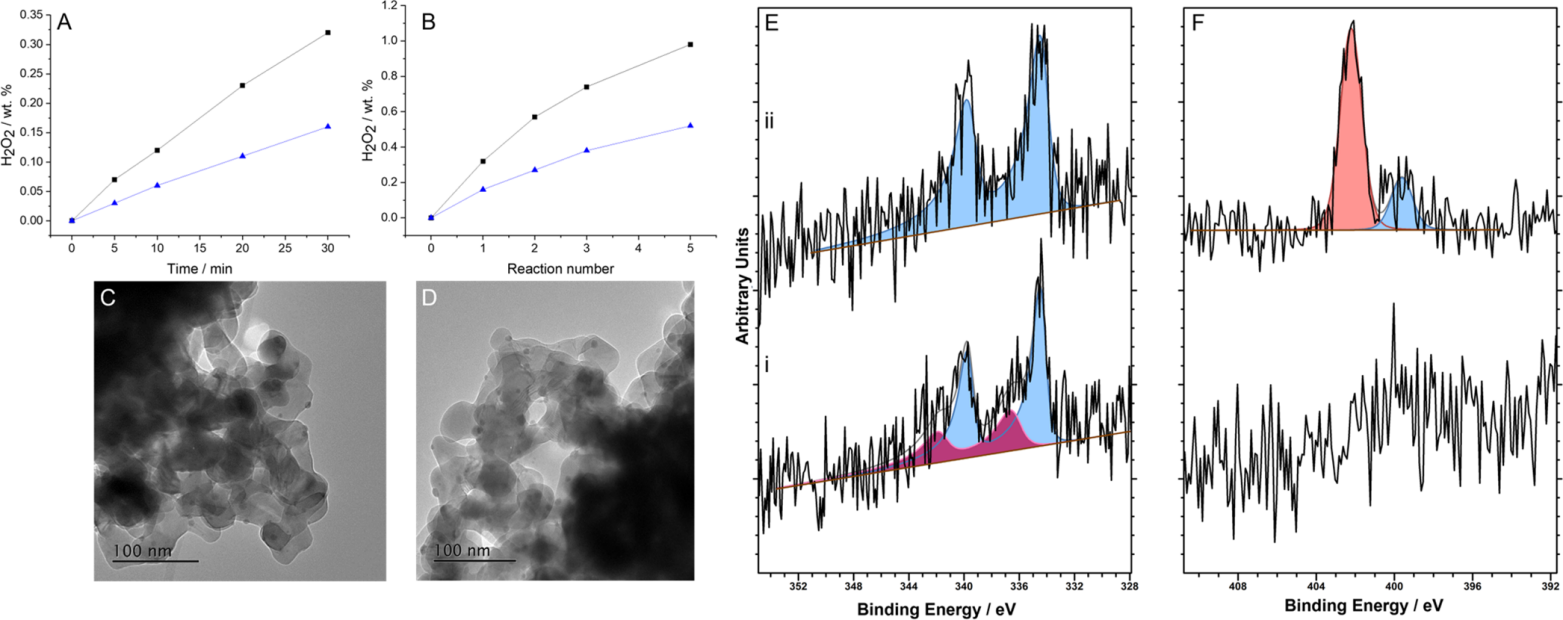
Comparison of catalytic performance toward the direct synthesis of H_2_O_2_ in addition to a structural and morphological analysis of the 1%Pd/TiO_2_ and 1%Pd-IPr(1:1)/TiO_2_ catalysts. (A) Catalytic activity as a function of reaction time and (B) over sequential H_2_O_2_ synthesis reactions. Key: 1%Pd/TiO_2_ (blue triangles); 1%Pd-IPr(1:1)/TiO_2_ (black squares). TEM micrographs of the (C) 1%Pd/TiO_2_ and (D) 1%Pd-IPr(1:1)/TiO_2_ catalysts. XPS spectra of (E) Pd(3d) and (F) N(1s) regions for (i) 1%Pd/TiO_2_ and (ii) 1%Pd-IPr(1:1)/TiO_2_ catalysts. Key: for the Pd 3d spectra Pd^2+^ (purple), Pd^0^ (blue); for the N 1s spectra, imidazolium salt (peach), NHC-Pd moiety (blue). H_2_O_2_ direct synthesis reaction conditions: catalyst (0.01g), H_2_O (2.9 g), MeOH (5.6 g), 5%H_2_/CO_2_ (420 psi), 25%O_2_/CO_2_ (160 psi), 0.5 h, 2 °C, 1200 rpm.

Numerous studies have demonstrated the strong relationship between catalytic activity toward H_2_O_2_ synthesis and the particle size of Pd-only catalysts.^44^ The determination of mean particle size via TEM (Figure 3C,D, with particle size distributions shown in Figure S.5) indicates no significant change as a result of the introduction of the IPr carbene onto the 1%Pd/TiO_2_ catalyst (mean particle sizes determined to be 2.0 and 2.4 nm for the 1%Pd/TiO_2_ and 1%Pd-IPr(1:1)/TiO_2_ catalysts, respectively). As such, it is reasonable to propose that the enhanced activity of the 1%Pd-IPr(1:1)/TiO_2_ catalyst is not associated with increased metal dispersion. However, an analysis of the 1%Pd-IPr(1:1)/TiO_2_ and 1%Pd/TiO_2_ catalysts via XPS indicated that the introduction of the IPr carbene leads to a significant shift in the Pd oxidation state, toward Pd^0^ (Figure 3E,F), corroborating our studies via CO-DRIFTS (Figure 2.B).

We further determined the high stability of both the 1%Pd/TiO_2_ and 1%Pd-IPr(1:1)/TiO_2_ catalysts. No loss in H_2_O_2_ synthesis activity was observed upon reuse of either material in the direct synthesis reaction (Table S.5), while ICP-MS analysis of post-reaction solutions (Table S.6) indicated negligible metal leaching over the course of a standard reaction. An analysis by TEM reveals a minor increase in mean particle size after use (Figure S.6), although such a shift occurs to a lesser extent over the NHC-incorporated material, while XPS (Figure S.7) reveals no significant variation in Pd oxidation state between the fresh and used materials. However, in the case of the IPr-modified sample, we do observe a substantial loss in the N(1s) signal associated with the residual parent imidazolium salt (centered at 403 eV), while the corresponding signal associated with IPr moiety interacting with Pd nanoparticles is retained, which correlates well with the observed stability of the catalytic material and indicates that the enhanced activity of the 1%Pd-IPr(1:1)/TiO_2_ catalyst results from the Pd–IPr interaction.

We have demonstrated the enhanced activity and selectivity of NHC-modified supported palladium nanoparticles toward the direct synthesis of H_2_O_2_. Our studies reveal the ability of the NHC ligands to act as electronic modifiers of Pd, in a way similar to that observed previously through the introduction of secondary metals, with the catalytic performance being retained upon reuse. Such results not only demonstrate the efficacy of these materials toward H_2_O_2_ formation but also highlight the role that this class of ligand may offer toward a range of heterogeneously catalyzed reaction pathways.

## Supporting Information Available

Experimental procedures, catalytic testing data, and material characterization (ATR-IR, XPS, CO-DRIFTS and TEM) in addition to metal leaching analysis via ICP-MS (PDF)

## Dedication

This paper is dedicated to Guy Bertrand on the occasion of his 70th birthday.
