# Supplementary material for: N-Heterocyclic Carbene Modified Palladium Catalysts for the Direct Synthesis of Hydrogen Peroxide
Source: J Am Chem Soc. 2022 Aug 17;144(34):15431–6. doi: 10.1021/jacs.2c04828 (PMC9449981; doi:10.1021/jacs.2c04828)
Supplement: Supplementary file 1 — ja2c04828_si_001.pdf [file ja2c04828_si_001.pdf]

Supporting Information

N-Heterocyclic Carbene Modified Palladium Catalysts for the Direct Synthesis of Hydrogen Peroxide

Richard J. Lewis[a‡], Maximilian Koy[b‡], Margherita Macino[a], Mowpriya Das[b], James H. Carter[a], David J. Morgan[a,c], Thomas E. Davies[a], Johannes B. Ernst[b], Simon J. Freakley[d], Frank Glorius[b,]* and Graham J. Hutchings[a,]*

[a]Max Planck Cardiff Centre on the Fundamentals of Heterogeneous Catalysis, FUNCAT, Cardiff Catalysis Institute, School of Chemistry, Cardiff University, Main Building, Park Place, Cardiff, CF103AT, United Kingdom.

[b]Westfälische Wilhelms-Universität Münster, Organisch-Chemisches Institut, Corrensstraße 40, 48149 Münster, Germany.

[c]Harwell XPS, Research Complex at Harwell (RcaH) Didcot, OX110FA, United Kingdom.

[d]Department of Chemistry, University of Bath, Claverton Down, Bath, BA27AY, United Kingdom.

General Methods

Chemical reagents used in catalyst preparation were purchased from Merck, Strem Chemicals and Evonik Industries and used as received. Solvents used for catalyst testing were purchased from Fischer Scientific as HPLC grade and used as received.

Experimental

Catalyst Preparation

A 1%Pd/TiO_2 catalyst has been prepared (on a weight basis) by an excess chloride wet-impregnation procedure, prior to NHC-modification, based on a methodology previously reported in the literature.[1] The procedure to produce 2 g of 1%Pd/TiO_2 is outlined below.

Aqueous acidified $PdCl_2$ solution (3.33 mL, 0.58 M HCl, 6 mgmL^{-1}, Sigma Aldrich) was heated to 65 °C with stirring (1000 rpm) in a thermostatically controlled oil bath in a 50 mL round bottom flask, with the total volume fixed to 16 mL using H_2O (HPLC grade). Upon reaching 65 °C TiO_2 (1.98 g, Degussa, P25) was added over the course of 5 minutes with constant stirring. The resulting slurry was stirred at 60 °C for a further 15 min, following this the temperature was raised to 95 °C for 16 h to allow for complete evaporation of water. The resulting solid was ground prior to heat treatment in a reductive atmosphere (flowing 5 % H_2 / Ar, 400 °C, 4h, 10 °C min^{-1}).

Prior to NHC modification, the 1%Pd/TiO_2 catalyst was first dried under vacuum (room temperature, 16 h). In a glovebox, the corresponding imidazolium salt (HBF_4 salts were used, only for IPr* the corresponding HCl salt was used) and KO*t*Bu (1.2 eq. of base per equivalent of imidazolium salt) were added to a Schlenk tube. Toluene (2 mL solvent/100 mg catalyst) was added under argon and the reaction mixture was allowed to stir at room temperature for 4 h. The reaction mixture was filtered under argon over a plug of Celite®. The plug was rinsed with additional toluene (2 mL solvent/100 mg catalyst). The solution of the free carbene was transferred to a Schlenk tube containing the 1%Pd/TiO_2 catalyst. The resulting suspension was stirred for 1 h at room temperature. Stirring was stopped and the solid catalyst was allowed to settle. The remaining solvent was removed by syringe and the catalyst was dried under vacuum (room temperature, 16 h).

Synthesis of Imidazolium Salts

Imidazolium salts were either commercially available or were prepared according to literature.[2] The synthesis of *p*Ph-IPr•HCl is shown below.

Synthesis of *p*Ph-IPr•HX

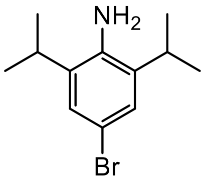

4-Bromo-2,6-diisopropylaniline was synthesized by following the literature procedure.[3] Br_2 (1.0 mL, 20.0 mmol) was dissolved in 7.5 mL of DCM and then added dropwise at 0 °C to a solution of 2,6-diisopropylaniline (4.0 mL, 20.0 mmol) in 100 mL of DCM. After the complete addition, the reaction was stirred for 30 min at room temperature. Then a saturated solution of $Na_2S_2O_3$ was added and stirred for another 15 min. The organic layer was separated, collected and concentrated. It was then extracted with 0.5 (M) NaOH and brine solution, the organic layer was dried over MgSO4 and concentrated under reduced pressure. Purification by column chromatography over silica with pentane/ethyl acetate afforded the product as a yellow oil.

^{1}H NMR (400 MHz, $CDCl_3$): δ (ppm) 7.12 (s, 2H), 3.72 (s, 2H), 3.01 – 2.78 (m, 2H), 1.33 – 1.20 (m, 12H).

^{13}C NMR (101 MHz, $CDCl_3$): δ (ppm) 139.4, 134.7, 125.9, 111.2, 28.1, 22.4.

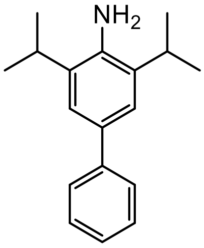

3,5-Diisopropyl-[1,1'-biphenyl]-4-amine was synthesized by following a literature known procedure. 4-bromo-2,6-diisopropylaniline (1.0 g, 3.9 mmol), phenylboronic acid (717 mg, 5.85 mmol) and $Pd(PPh_3)_4$ (225 mg, 0.195 mmol) were taken in a Schlenk flask and toluene (25 mL) was added to the mixture. To this solution, a deoxygenated solution of Na_2CO_3 (10 mL, 2 M solution in water) mixed with ethanol (6.5 mL) was added. The biphasic reaction mixture was then refluxed for 48 h. After cooling the reaction, the organic layer was separated and filtered through a pad of Celite and the solvent evaporated and purified the product by flash

column chromatography (pentane : ethyl acetate = 25 : 1). The product was obtained as a white solid (889 mg, 3.5 mmol, 90 % yield).

^{1}H NMR (400 MHz, $CDCl_3$): δ (ppm) 7.59 – 7.50 (m, 2H), 7.42 – 7.33 (m, 2H), 7.29 – 7.19 (m, 3H), 3.77 (s, 2H), 3.06 – 2.81 (m, 2H), 1.33 – 1.28 (m, 12H).

^{13}C NMR (101 MHz, $CDCl_3$): δ (ppm) 142.5, 140.0, 132.8, 131.6, 128.7, 126.8, 126.2, 121.9, 28.2, 22.6.

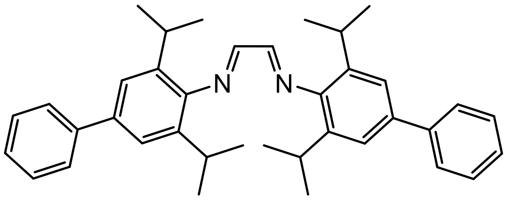

N^1,N^2-Bis(3,5-diisopropyl-[1,1'-biphenyl]-4-yl)ethane-1,2-diimine: To a solution of 3,5-diisopropyl-[1,1'-biphenyl]-4-amine (1.0 g, 4.0 mmol) in methanol (3 mL), glyoxal solution (0.3 mL, 2.4 mmol, 40 % in water) was added dropwise, at room temperature. After the addition, 2 drops of acetic acid were added to the mixture and the reaction was stirred overnight. A yellow precipitate was formed and it was washed with cold methanol (2 mL). The product was used for the next step without any further purification.

^{1}H NMR (400 MHz, $CDCl_3$): δ (ppm) 8.02 (s, 2H), 7.50 – 7.45 (m, 4H), 7.43 – 7.24 (m, 6H), 7.23 – 7.08 (m, 4H), 2.94 – 2.81 (m, 4H), 1.15 – 1.09 (m, 24H).

^{13}C NMR (101 MHz, $CDCl_3$): δ (ppm) 163.4, 147.5, 141.8, 138.2, 137.4, 128.9, 127.2, 127.1, 122.4, 28.4, 23.6.

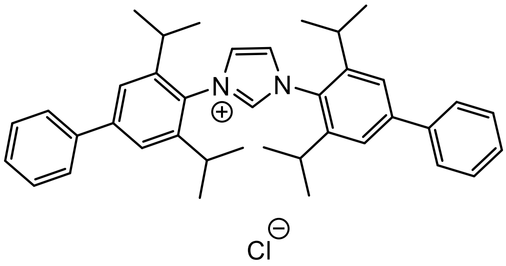

1,3-Bis(3,5-diisopropyl-[1,1'-biphenyl]-4-yl)-1H-imidazol-3-ium chloride: N^{1},N^2-bis(3,5-diisopropyl-[1,1'-biphenyl]-4-yl)ethane-1,2-diimine (950 mg, 1.80 mmol) and $ZnCl_2$ (245 mg, 1.80 mmol) were stirred for 5 min at 70 °C in dry THF (70 mL). Paraformaldehyde (59.4 mg, 1.98 mmol) and HCl (0.45 mL, 4 M in dioxane) were added to the reaction mixture and it was stirred for 3 h at 70 °C. The reaction mixture was cooled to room temperature and all the volatiles were removed in vacuuo. The residue was dissolved in ethyl acetate and washed with water and brine solution. After that, the aqueous phase was extracted with ethyl acetate.

All the organic layers were combined, dried over Na_2SO_4, filtered and concentrated. Then the residue was dissolved in DCM and washed with diethyl ether. The product was obtained as white solid (560 mg, 0.97 mmol, 54 % yield). Anion exchange from chloride to tetrafluoroborate: The corresponding chloride salt (1.0 eq) was dissolved in CH_2Cl_2 and $AgBF_4$ (1.2 eq.) was added in one portion. The suspension was filtered over celite and concentrated. The crude product was further purified by silica gel chromatography (CH_2Cl_2:MeOH), dried under vacuum and used for catalyst modification.

^{1}H NMR (400 MHz, $CDCl_3$): δ (ppm) 10.44 (s, 1H), 8.15 – 8.10 (m, 2H), 7.61 – 7.54 (m, 4H), 7.51 – 7.45 (m, 8H), 7.44 – 7.38 (m, 2H), 2.55 – 2.42 (m, 4H), 1.35 – 1.24 (m, 24H). **^{13}C NMR** (101 MHz, $CDCl_3$): δ (ppm) 145.4, 145.2, 140.2, 139.4, 129.2, 129.1, 128.4, 127.5, 126.8, 123.7, 29.4, 24.9, 23.8. **HRMS (ESI)**: m/z calculated for $C_{39}H_{45}N_2$ [M-Cl]$^+$: 541.3577; found: 541.3584.

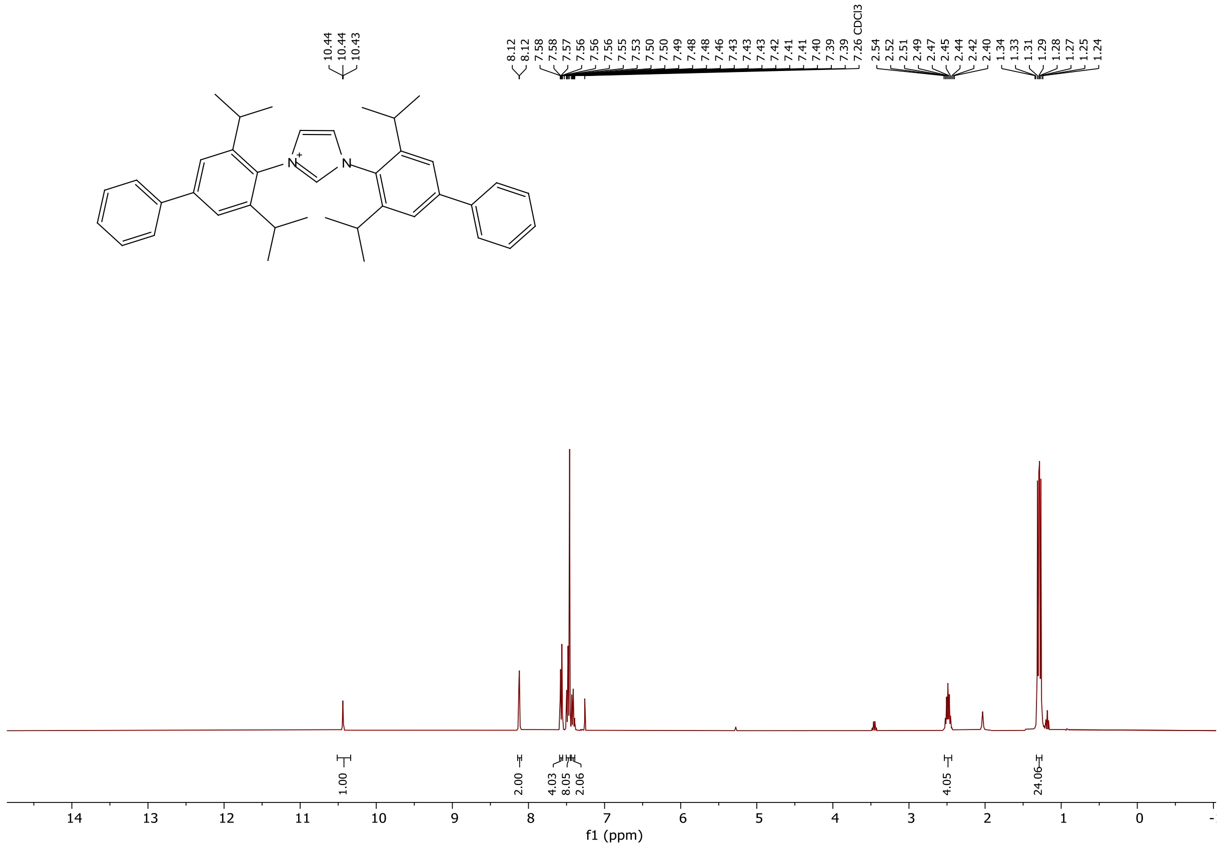
10.44
10.44
10.43
8.12
8.12
7.58
7.57
7.56
7.56
7.55
7.53
7.50
7.50
7.49
7.48
7.48
7.46
7.43
7.43
7.43
7.42
7.41
7.41
7.40
7.39
7.39
7.26 CDCl3
2.54
2.52
2.51
2.49
2.47
2.45
2.44
2.42
2.40
1.34
1.33
1.31
1.29
1.28
1.27
1.25
1.24
1.00
2.00
4.03
8.05
2.06
4.05
24.06
14
13
12
11
10
9
8
7
6
5
4
3
2
1
0
f1 (ppm)

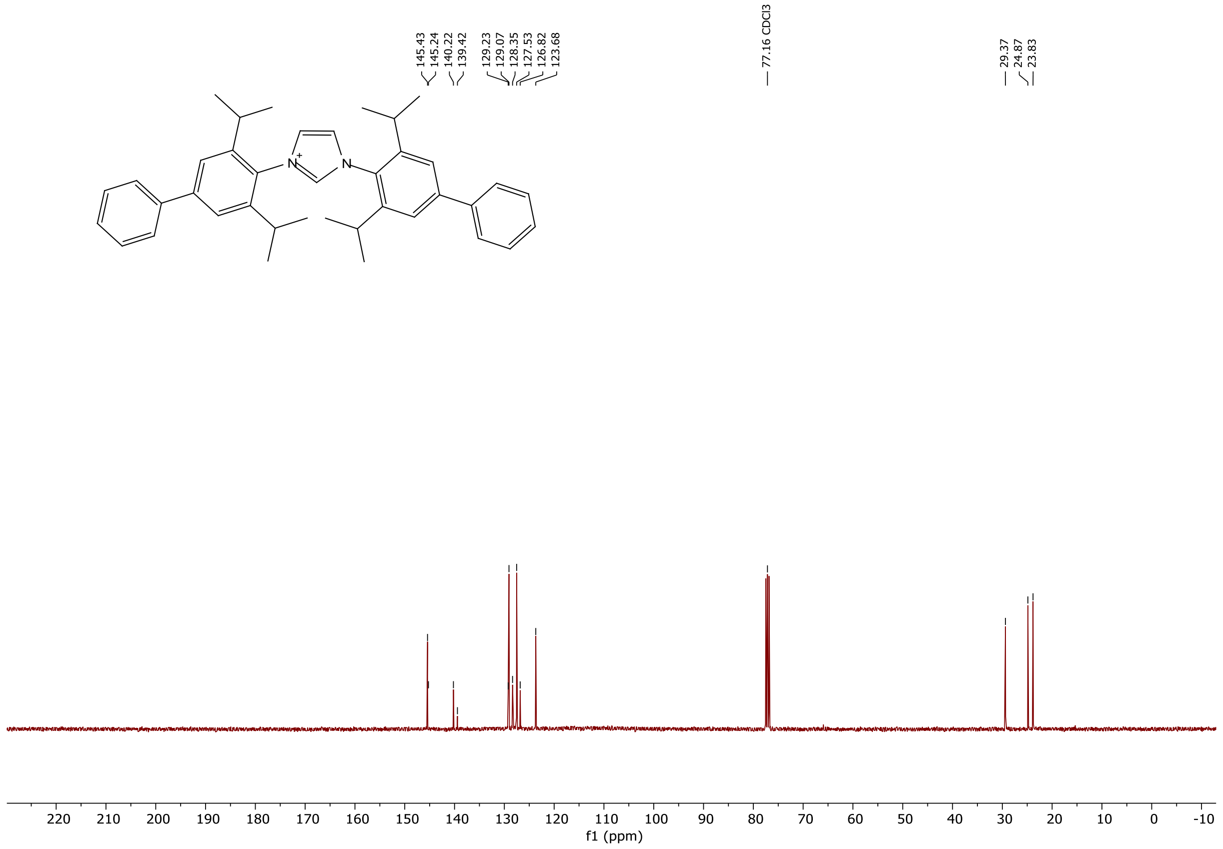
145.43
145.24
140.22
139.42
129.23
129.07
128.35
127.53
126.82
123.68
77.16 CDCl3
29.37
24.87
23.83
220
210
200
190
180
170
160
150
140
130
120
110
100
90
80
70
60
50
40
30
20
10
0
-10
f1 (ppm)

Catalyst Testing

Note 1: Reaction conditions used within this study operate below the flammability limits of gaseous mixtures of H_2 and O_2. In all reactions, reactor temperature was controlled using a HAAKE K50 bath/circulator using an appropriate coolant. The reactor temperature was maintained at 2 °C ±0.2°C throughout the course of the H_2O_2 synthesis and degradation reaction.

Note 2: The conditions used within this work for H_2O_2 synthesis and degradation have previously been investigated, with the presence of CO_2 as a diluent for reactant gases and a methanol co-solvent identified as key to maintaining high catalytic efficacy towards H_2O_2 production.[4]

Note 3: In all cases, reactions were run multiple times with the data presented as an average of these experiments. Catalytic activity towards H_2O_2 synthesis was found to be consistent to within ± 2% based on multiple batches of catalyst and multiple reactions.

Direct synthesis and degradation of H_2O_2

Hydrogen peroxide synthesis activity was evaluated using a Parr Instruments stainless steel autoclave with a nominal volume of 100 mL and a maximum working pressure of 2000 psi. To test each catalyst for H_2O_2 synthesis, the autoclave was charged with catalyst (0.01 g) and solvent (5.6 g methanol and 2.9 g H_2O, both HPLC grade, Fischer Scientific). The charged autoclave was then purged three times with 5% H_2/CO_2 (100 psi) before filling with 5% H_2/CO_2 to a pressure of 420 psi, followed by the addition of 25% O_2/CO_2 (160 psi). The reactor was not continually supplied with gas. The reaction was cooled to a temperature of 2 °C, prior to stirring (1200 rpm) for 30 min. After 30 min, the stirring was ceased, the reactant gas was sampled, and the catalyst was removed from the reaction solution via filtration. H_2O_2 productivity was determined by titrating aliquots of the final solution after reaction with acidified $Ce(SO_4)_2$ (0.0085 M) in the presence of ferroin indicator. Catalyst productivities are reported as $mol_{H2O2}kg_{cat}^{-1}h^{-1}$.

Additional direct synthesis experiments were conducted under reaction conditions considered less conducive toward H_2O_2 stability, namely removing the methanol co-solvent, replacing the CO_2 gaseous reagent diluent with an inert alternative (N_2), and utilising ambient reaction temperatures (20 °C). The use of CO_2 as a gaseous diluent, under identical reaction conditions to those used within this work, has previously been reported to reduce reaction solution pH (to a value of pH 4) and result in a comparable improvement in catalytic activity as observed through acidification with HNO_3.[5]

Total autoclave capacity was determined via water displacement to allow for accurate determination of H_2 conversion and H_2O_2 selectivity. When equipped with a PTFE liner, the total volume of an unfilled autoclave was determined to be 93 mL, which includes all available gaseous space within the autoclave.

Catalytic conversion of H_2 and selectivity towards H_2O_2 were determined using a Varian 3800 GC fitted with TCD and equipped with a Porapak Q column.

H_2 conversion (Equation 1) and H_2O_2 selectivity (Equation 2) are defined as follows:

$$\mathrm{H_2Conversion\ (\%)} = \frac{\mathrm{mmol_{H2\ (t(0))}} - \mathrm{mmol_{H2\ (t(1))}}}{\mathrm{mmol_{H2\ (t(0))}}} \times\ 100\ (eq\ 1\ )$$

$$\mathrm{H_2O_2\ Selectivity\ (\%)} = \frac{\mathrm{H_2O_2 detected\ (mmol)}}{\mathrm{H_2\ consumed\ (mmol)}} \times 100\ \ (eq\ 2)$$

Catalytic activity towards total H_2O_2 degradation (the sum of both hydrogenation and decomposition pathways) was determined in a manner similar to the direct synthesis activity of H_2O_2. The autoclave was charged with catalyst (0.01 g), solvent (methanol (5.6 g) and H_2O (2.21 g) both HPLC grade, Fischer Scientific) and H_2O_2 (50 wt.% 0.69 g, Merck), with the solvent composition equivalent to a 4 wt. % H_2O_2 solution. From the solution two aliquots of 0.05 g were removed and titrated with acidified $Ce(SO_4)_2$ solution using ferroin as an indicator to determine an accurate concentration of H_2O_2 at the start of the reaction. The autoclave was purged with 5%H_2/CO_2 (100 psi) prior to being pressurised with 5% H_2/CO_2 (420 psi). The reaction was conducted at a temperature of 2 °C, for 30 min with stirring (1200 rpm). After the reaction was complete the catalyst was removed from the reaction solvents and as previously two aliquots of 0.05 g were titrated against the acidified $Ce(SO_4)_2$ solution using ferroin as an indicator.

Gas replacement experiments for the direct synthesis of H_2O_2

An identical procedure to that outlined above for the direct synthesis of H_2O_2 was followed for a reaction time of 30 min. After this, stirring was stopped and the reactant gas mixture was vented prior to replacement with the standard pressures of 5% H_2/CO_2 (420 psi) and 25% O_2/CO_2 (160psi). The reaction mixture was then stirred (1200 rpm) for a further 30 min. To collect a series of data points, as in the case of Figure 2.B, it should be noted that individual experiments were carried out and the reactant mixture was not sampled on-line.

Catalyst reusability in the direct synthesis and degradation of H_2O_2

In order to determine catalyst reusability a similar procedure to that outlined above for the direct synthesis of H_2O_2 is followed utilising 0.05 g of catalyst. Following the initial test, the catalyst was recovered by filtration and dried (30 °C, 16 h, under vacuum). From the recovered catalyst sample 0.01 g was subsequently used to conduct a standard H_2O_2 synthesis or degradation test.

Catalyst characterisation

X-ray photoelectron spectroscopy (XPS) analyses were performed on a Kratos Axis Ultra DLD spectrometer. Samples were mounted using double-sided adhesive tape and binding energies were referenced to the C (1s) binding energy of the lowest carbon species taken to be 285.0 eV. Monochromatic AlK_{α} radiation was used for all measurements; an analyser pass energy of 160 eV was used for survey scans while 40 eV was employed for detailed regional scans. The intensities of the Pd ($3d_{5/2}$) and Pd($3d_{3/2}$) in the 334-340 and 340-346 eV regions were used to derive the Pd(II) : Pd(0) surface ratios. Analysis of catalytic samples, after use in the direct synthesis of H_2O_2 was conducted after the sample was dried under vacuum (30 °C, 16h). It was noted that under relatively short times frames, the carbenes were sensitive to analysis induced reduction (see Figure S.2), therefore the data were acquired in an optimised time frame to minimise such reduction but still with a workable signal-to-noise ratio to allow quantification and peak fitting where required.

Transmission electron microscopy (TEM) was performed on a JEOL JEM-2100 operating at 200 kV. Samples were prepared by dispersion in ethanol by sonication and deposited on 300 mesh copper grids coated with holey carbon film. Energy dispersive X-ray analysis (EDX) was performed using an Oxford Instruments X-Max^{N} 80 detector and the data analysed using the Aztec software.

Metal leaching during the direct synthesis reaction was quantified using an Agilent 7900 ICP-MS equipped with an I-AS auto-sampler using a 5-point calibration using certified reference materials from Perkin Elmer and certified internal standard from Agilent. All calibrants were matrix matched.

Attenuated Total Reflectance-Infrared (ATR-IR) spectra of the solid samples was gathered using a Bruker Platinum ATR Vertex 70. Transmittance was measured between 4000 and 500 cm^{-1} with a resolution of 2 cm^{-1} using a MIR source, a Liquid Nitrogen cooled Mercury Cadmium Telluride (LN-MCT) detector and a KBr beam splitter.

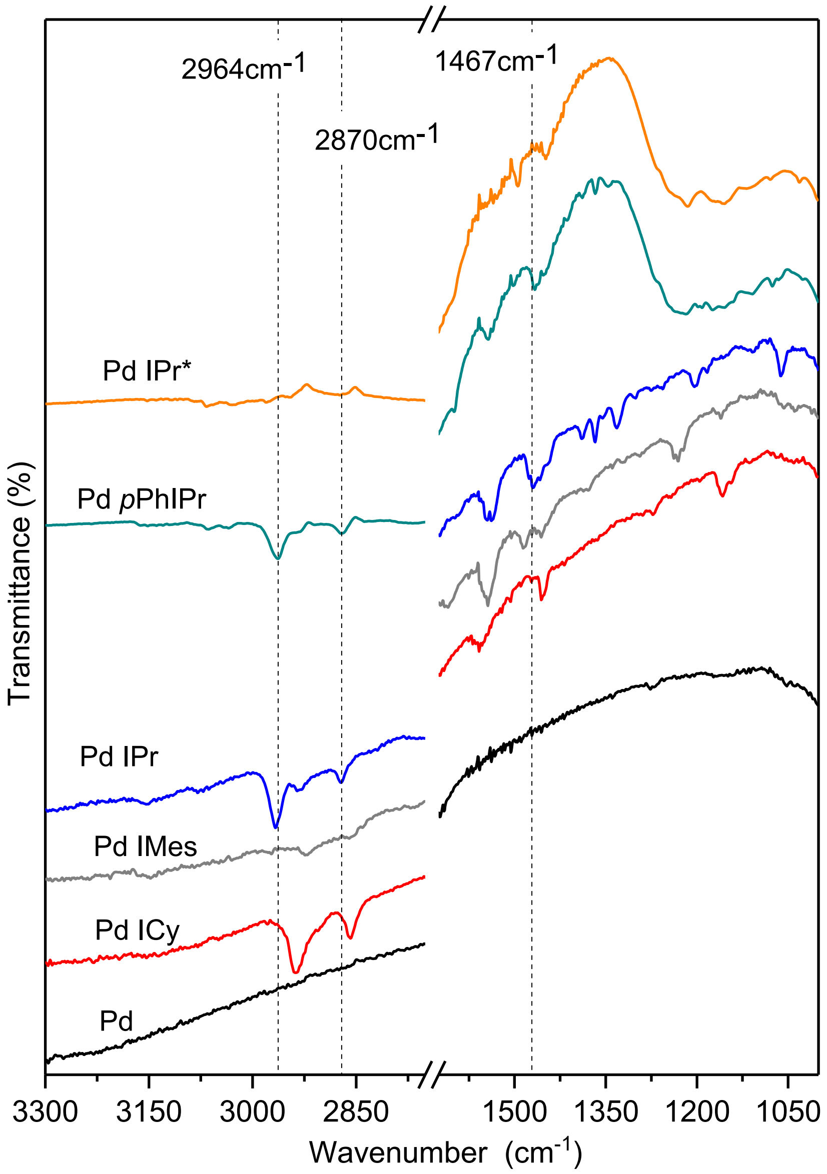

Figure S.1. Attenuated Total Reflectance-Infrared (ATR-IR) spectra of NHC-modified 1%Pd/TiO_2 catalyst. **Note:** Our observations via ATR-IR are in keeping with previous studies by Ranganath *et al.* [6]who have reported the characteristic NHC absorption bands to be between approximately 2850-3000 cm^{-1} and 1100-1550 cm^{-1}.

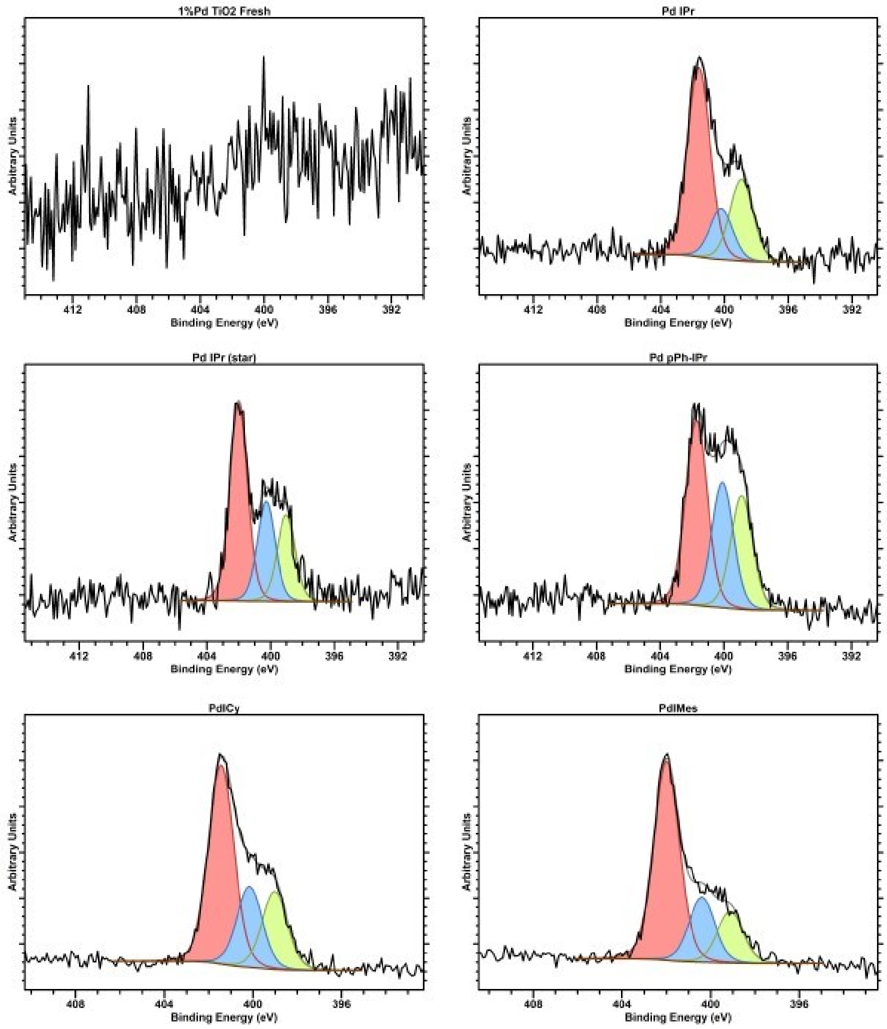

Figure S.2. XPS analysis of the N(1s) region of N-heterocyclic carbene modified 1%Pd/TiO_2 catalysts. Fitting of the signal indicates 3 species, the first (red) is attributed to the carbene, the blue species is that of carbene associated with the Pd and the green that as a product of reduction from prolonged XPS analysis. Note these spectra are acquired after longer acquisition times than those reported elsewhere in this work, hence the notable reduced species (see experimental section above).

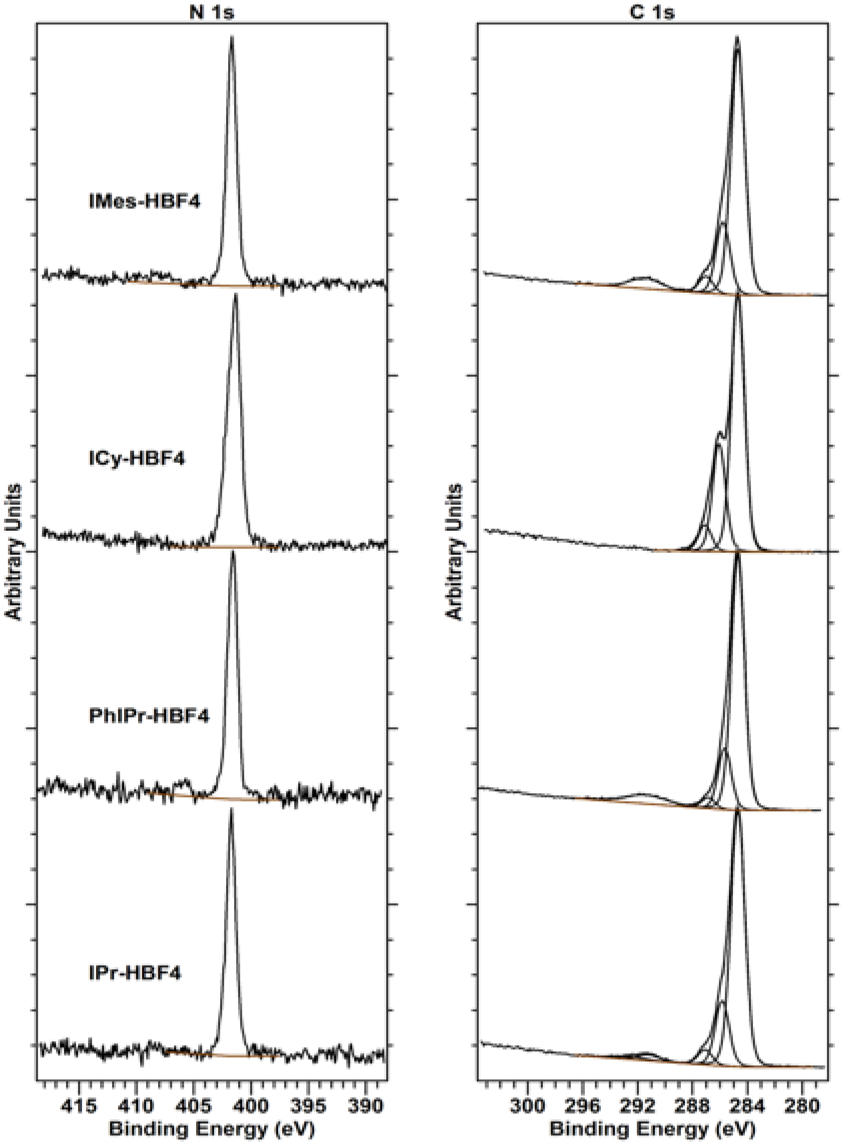

Figure S.3. XPS analysis of the N (1s) and C (1s) regions of the parent imidazolium salts prior to their deposition on to the 1%Pd/TiO_2 catalysts.

Table S.1. Catalytic activity comparisons against state-of-the-art catalysts for the direct synthesis of H_2O_2.

Catalyst	Productivity / $mol_{H2O2}kg_{cat}^{-1}h^{-1}$	H_2O_2 Conc. / wt.%	H_2O_2 Sel. / %	Reference
1%Pd/TiO_2	80	0.16	22	This Work
1%Pd-IPr(1:1)/TiO_2	160	0.32	64	This Work
0.5%Au-0.5%Pd/TiO_2	85	0.17	54	7
3%Pd-2%Sn/TiO_2	61	0.11 (0.53)	96	8
2.5%Au-2.5%Pd/C (2%HNO_3)	160	0.32 (1.09)	>98	9

H_2O_2 direct synthesis reaction conditions*:* Catalyst (0.01g), H_2O (2.9g), MeOH (5.6g), 5% H_2 / CO_2 (420 psi), 25% O_2 / CO_2 (160 psi), 0.5 h, 2 ˚C 1200 rpm.

Table S.2. The role of gaseous reagent diluent and reaction solution acidification on catalytic performance towards the direct synthesis of H_2O_2.

Catalyst	Diluent	Productivity / $mol_{H2O2}kg_{cat}^{-1}h^{-1}$	H_2O_2 Conc. / wt.%
1%Pd/TiO_2	CO_2	80	0.16
	N_2	34	0.07
1%Pd-IPr(1:1)/TiO_2	CO_2	160	0.32
	N_2	85	0.17

H_2O_2 direct synthesis reaction conditions*:* Catalyst (0.01g), H_2O (2.9g), MeOH (5.6g), 5% H_2 / CO_2 or 5% H_2 / N_2 (420 psi), 25% O_2 / CO_2 or 25% O_2 / N_2 (160 psi), 0.5 h, 2 ˚C 1200 rpm. **Note:** The CO_2 gaseous diluent has been previously identified to result in a considerable reduction in reaction solution pH, with a resulting enhancement in catalytic activity comparable to that observed when utilising HNO_3 as an exogenous additive.[5]

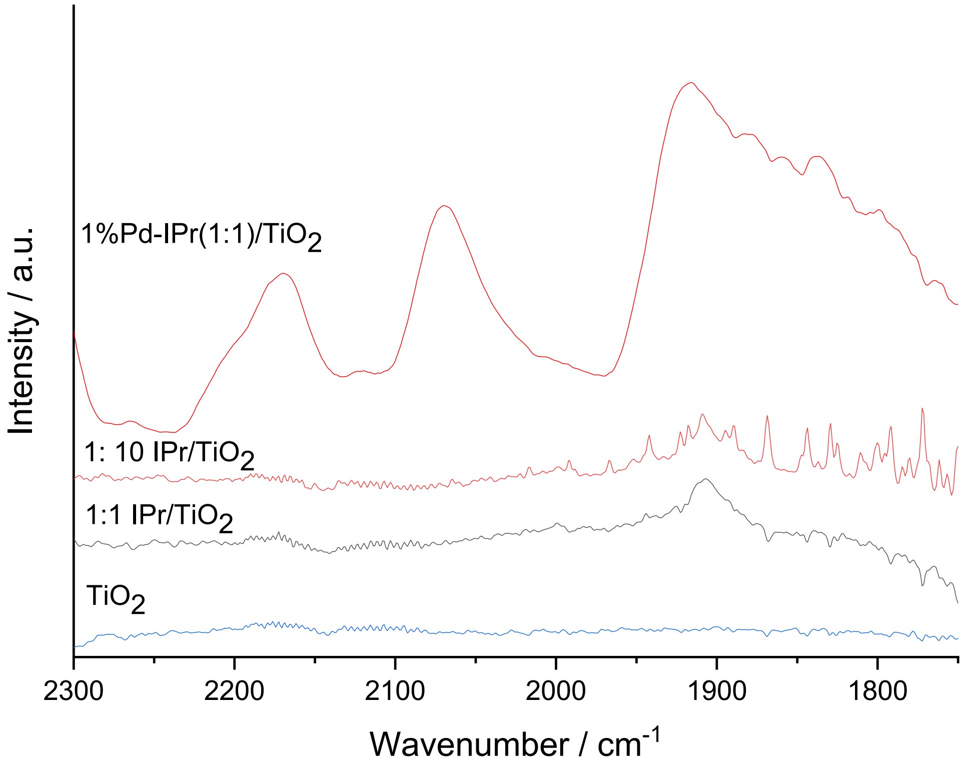

Figure S.4. CO-DRIFTS spectra of the IPr/TiO_2 material, in the presence and absence of the Pd nanoparticles. **Note:** The feature at approximately 1900 cm^{-1}.

Supplementary note for Figure S.4

The CO-DRIFTS spectrum of IPr/TiO_2 material (*i.e.* without Pd present) can be seen to present a feature at 1900 cm^{-1}, suggesting some interaction of CO with the NHC ligand or residual imidazolium salt. In the case of the 1%Pd/TiO_2 catalyst, features at 1900 cm^{-1} are observed, which suggest an overlap of CO-Pd and CO-NHC/imidazolium salt interactions in this region of the spectrum.

Table S.3. Comparison of catalytic selectivity towards H_2O_2 and H_2 conversion.

Catalyst	H_2 Conv. / %	H_2O_2 Sel. / %	Productivity/ $mol_{H2O2}kg_{cat}^{-1}h^{-1}$	H_2O_2 Conc. / wt.%
1% Pd / TiO_2	35	22	80	0.16
1%Pd-IPr(1:1)/TiO_2	25	64	160	0.32

H_2O_2 direct synthesis reaction conditions*:* Catalyst (0.01g), H_2O (2.9g), MeOH (5.6g), 5% H_2 / CO_2 (420 psi), 25% O_2 / CO_2 (160 psi), 0.5 h, 2 ˚C 1200 rpm.

Table S.4. XPS derived surface atomic concentrations of as-prepared 1%Pd/TiO_2 and 1%Pd-IPr(1:1)/TiO_2 catalysts and after use in the direct synthesis of H_2O_2.

Catalyst	Surface atom / %						
	O 1s	C 1s	N 1s	F 1s	Ti 2p	Cl 2p	Pd 3d
1% Pd / TiO_2 (Fresh)	34.4	55.3	0	0.3	9.7	0.1	0.1
1% Pd / TiO_2 (Used)	34.2	56.2	0	0.2	9.2	0.1	0.1
1%Pd-IPr(1:1)/TiO_2 (Fresh)	36.1	49.6	0.8	0.7	12.5	0.2	0.1
1%Pd-IPr(1:1)/TiO_2 (Used)	46.8	34.2	0.5	0.4	17.9	0.1	0.1

Supplementary note for Table S.4

While no significant variation in surface Cl levels were observed across this subset of materials, the surface concentration of F was found to be higher in the as-prepared 1%Pd-IPr(1:1)/TiO_2 catalyst, with this attributed to the presence of residual parent imidazolium salt. While this may be considered the underlying cause for the improved catalytic performance of this catalyst, with halides known to often promote H_2O_2 selectivity, it is important to note that the activity of the catalyst was retained upon re-use (Table S.4), despite the corresponding loss in surface F concentrations. Additionally, further experiments (Table 1), reveals that a physical mixture of 1%Pd/TiO_2 and imidazolium offers no greater catalytic activity towards H_2O_2 synthesis (79 $mol_{H2O2}kg_{cat}^{-1}h^{-1}$) than the 1%Pd/$TiO_2$ catalyst alone (80 $mol_{H2O2}kg_{cat}^{-1}h^{-1}$). As such it is possible to rule out any contribution from residual halide to the observed catalysis.

Table S.5. Comparison of catalytic performance upon first and second use in the H_2O_2 direct synthesis reaction.

	First Use		Second Use*	
**Catalyst**	**Productivity / $mol_{H2O2}kg_{cat}^{-1}h^{-1}$****	**Initial reaction rate / $mmol_{H2O2}min^{-1}$**	**Productivity / $mol_{H2O2}kg_{cat}^{-1}h^{-1}$****	**Initial reaction rate / $mmol_{H2O2}min^{-1}$**
1% Pd / TiO_2	80	1.5x10^{-3}	80	1.5x10^{-3}
1%Pd-IPr(1:1)/TiO_2	160	3.5x10^{-3}	160	3.5x10^{-3}

*Catalyst used for 30 min under standard reaction conditions prior to determination of reaction rate over 0.083 or 0.5 h. **Productivity as determined over standard reaction time (0.5 h).**H_2O_2 direct synthesis reaction conditions**: Catalyst (0.01g), H_2O (2.9g), MeOH (5.6g), 5% H_2 / CO_2 (420 psi), 25% O_2 / CO_2 (160 psi), 2 ˚C 1200 rpm. **Note:** Initial rate of reaction determined based on nominal metal loading and over 0.083 h.

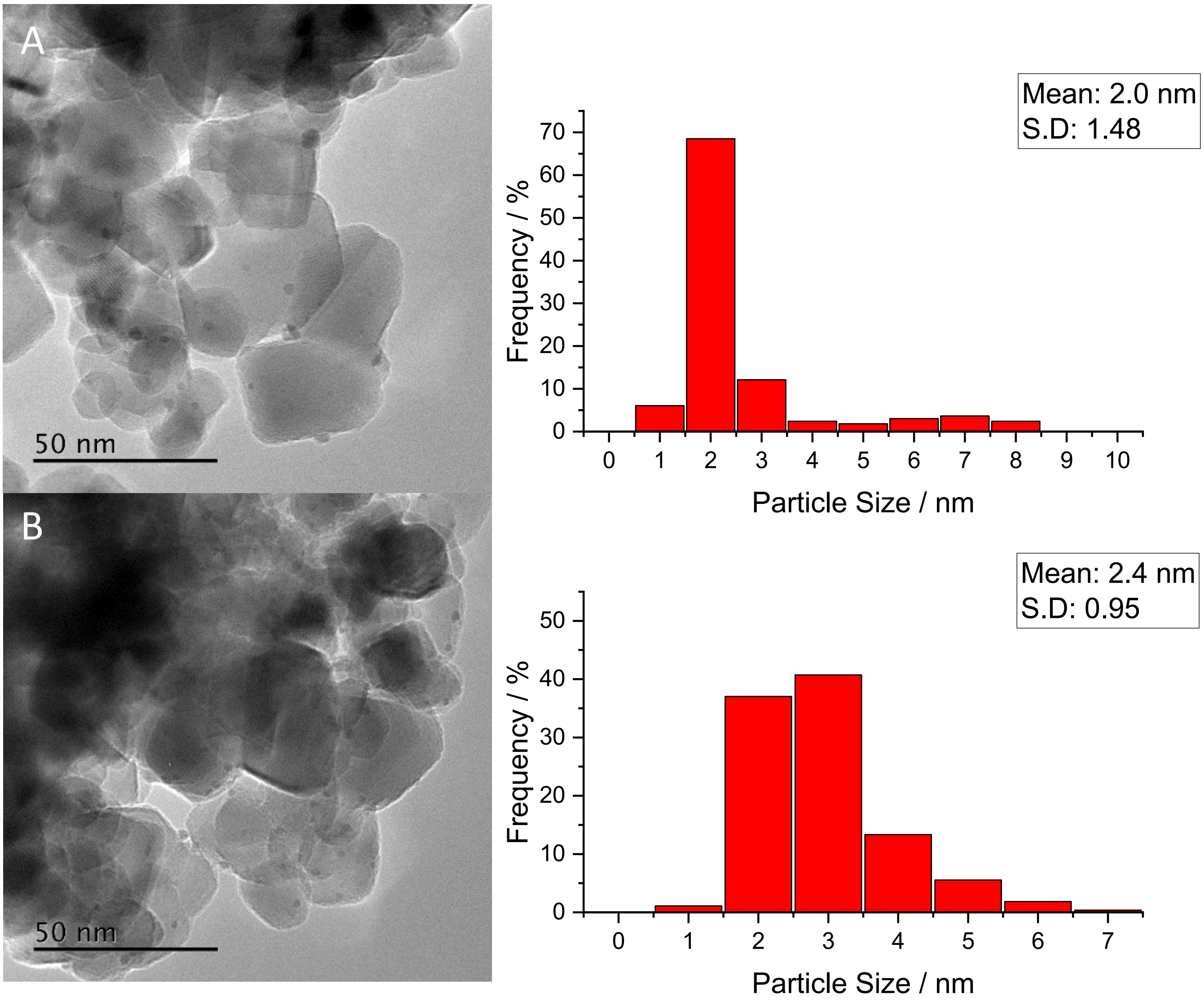

Figure S.5. Transmission electron microscopy of fresh **(A)** 1%Pd/TiO_2 and **(B)** 1%Pd-IPr(1:1)/TiO_2 catalysts.

Table S.6. Total metal leaching during H_2O_2 direct synthesis reaction, as determined by ICP-MS analysis of post-reaction solution.

Catalyst	Productivity (Fresh) / $mol_{H2O2}kg_{cat}^{-1}h^{-1}$	Productivity (Used) / $mol_{H2O2}kg_{cat}^{-1}h^{-1}$	Pd Leaching / %
1% Pd / TiO_2	80	80	0.02
1%Pd-IPr(1:1)/TiO_2	160	160	0.09

H_2O_2 direct synthesis reaction conditions: Catalyst (0.01g), H_2O (2.9g), MeOH (5.6g), 5% H_2 / CO_2 (420 psi), 25% O_2 / CO_2 (160 psi), 0.5 h, 2 ˚C 1200 rpm.

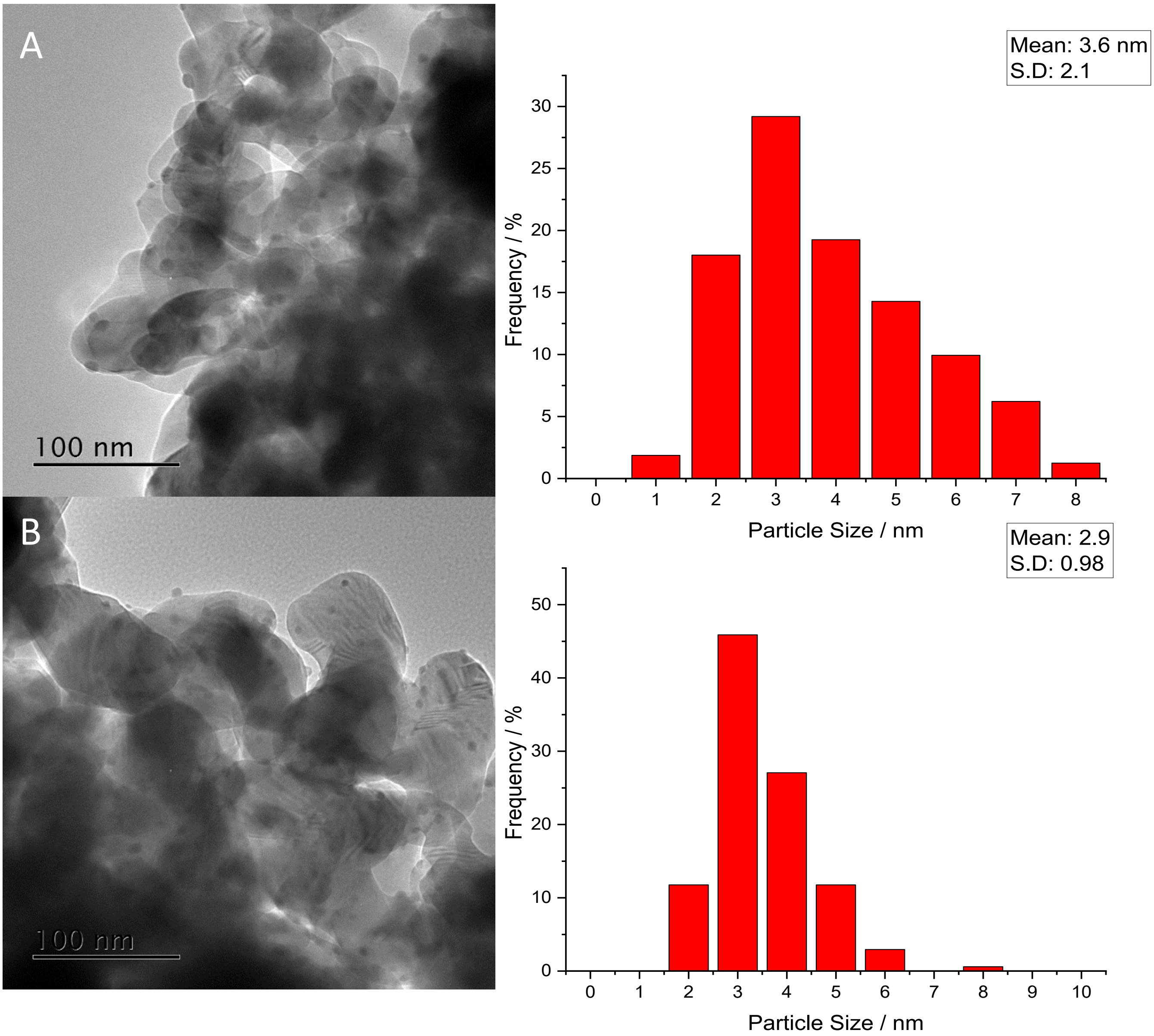

Figure S.6 Transmission electron microscopy of **(A)** 1%Pd/TiO_2 and **(B)** 1%Pd-IPr(1:1)/TiO_2 catalysts after use in the direct synthesis of H_2O_2.

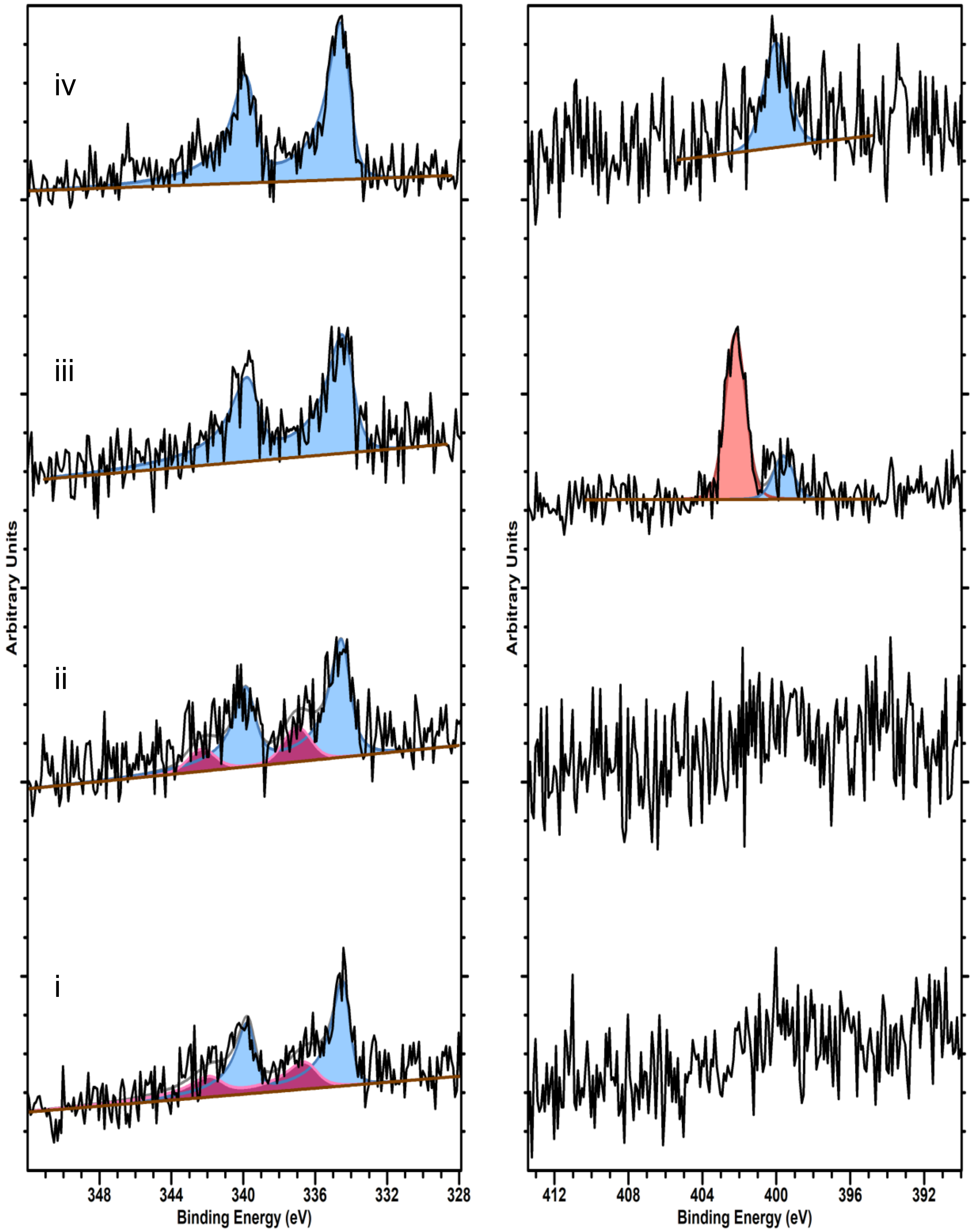

Figure S.7. XPS spectra of Pd(3d) and N(1s) regions for fresh **(i)** and **(iii)** and used **(ii)** and **(iv)** 1%Pd/TiO_2 and 1%Pd-IPr(1:1)/TiO_2 catalysts. ***Key:*** For the Pd 3d spectra purple is Pd^{2+} and blue is Pd^0. For N (1s) spectra, pink is NHC salt, and blue NHC interacting with Pd. **Note:** The intensity of the spectra presented are low due to the rapid acquisition times used to minimise the carbene degradation as discussed in the experimental section.

References

1. Sankar, M.; He, Q.; Morad, M.; Pritchard, J.; Freakley, S. J.; Edwards, J. K.; Taylor, S. H.; Morgan, D. J.; Carley, A. F.; Knight, D. W.; Kiely, C. J.; Hutchings, G. J., Synthesis of stable ligand-free gold-palladium nanoparticles using a simple excess anion method. *ACS Nano* **2012**, *6* (8), 6600-6613.
2. Hans, M.; Lorkowski, J.; Demonceau, A.; Delaude, L., Efficient synthetic protocols for the preparation of common N-heterocyclic carbene precursors. *Beilstein J. Org. Chem.* **2015**, *11*, 2318-2325.
3. Koehne, I.; Graw, N.; Teuteberg, T.; Herbst-Irmer, R.; Stalke, D., Introducing NacNac-Like Bis(4,6-isopropylbenzoxazol-2-yl)methanide in s-Block Metal Coordination. *Inorg. Chem.* **2017**, *56* (24), 14968-14978.
4. Santos, A.; Lewis, R. J.; Malta, G.; Howe, A. G. R.; Morgan, D. J.; Hampton, E.; Gaskin, P.; Hutchings, G. J., Direct Synthesis of Hydrogen Peroxide over Au–Pd Supported Nanoparticles under Ambient Conditions. *Ind. Eng. Chem. Res.* **2019**, *58* (28), 12623-12631.
5. Edwards, J. K.; Thomas, A.; Carley, A. F.; Herzing, A. A.; Kiely, C. J.; Hutchings, G. J., Au–Pd supported nanocrystals as catalysts for the direct synthesis of hydrogen peroxide from H_2 and O_2. *Green Chem.* **2008**, *10* (4), 388-394.
6. Ranganath, K. V. S.; Kloesges, J.; Schäfer, A. H.; Glorius, F., Asymmetric Nanocatalysis: N-Heterocyclic Carbenes as Chiral Modifiers of Fe_3O_4/Pd nanoparticles. *Angew. Chem. Int. Ed.* **2010**, *49*, 7786-7789.
7. Richards, T.; Lewis, R. J.; Morgan, D. J.; Hutchings, G. J., The Direct Synthesis of Hydrogen Peroxide Over Supported Pd-Based Catalysts: An Investigation into the Role of the Support and Secondary Metal Modifiers. *Catalysis Letters* **2022**. doi.org/10.1007/s10562-022-03967-8.
8. Freakley, S. J.; He, Q.; Harrhy, J. H.; Lu, L.; Crole, D. A.; Morgan, D. J.; Ntainjua, E. N.; Edwards, J. K.; Carley, A. F.; Borisevich, A. Y.; Kiely, C. J.; Hutchings, G. J., Palladium-tin catalysts for the direct synthesis of H_2O_2 with high selectivity. *Science* **2016**, *351*, 965-968.
9. Edwards, J. K.; Solsona, B.; Ntainjua, E. N.; Carley, A. F.; Herzing, A. A.; Kiely, C. J.; Hutchings, G. J., Switching off hydrogen peroxide hydrogenation in the direct synthesis process. *Science* **2009**, *323*, 1037-1041.
